# Carers’ views on autism and eating disorders comorbidity: qualitative study

**DOI:** 10.1192/bjo.2020.36

**Published:** 2020-05-18

**Authors:** James Adamson, Emma Kinnaird, Danielle Glennon, Madeleine Oakley, Kate Tchanturia

**Affiliations:** Department of Psychological Medicine, Kings College London, UK; National Eating Disorder Service, South London and Maudsley NHS Foundation Trust, UK; National Eating Disorder Service, South London and Maudsley NHS Foundation Trust, UK; and Department of Psychological Medicine, Kings College London, UK

**Keywords:** Families, carers’ needs, autism, eating disorders, treatment adaptation

## Abstract

**Background:**

Patients with co-occurring anorexia nervosa and autism respond differently to eating disorder treatments. Previous interviews with patients with both conditions and clinicians working in eating disorder services has highlighted service and treatment adaptations might be beneficial and could improve outcomes for these individuals.

**Aims:**

The aim of this study was to explore carers’ experiences of current treatment approaches for people with autism who have anorexia nervosa, and their views on how these can be improved.

**Method:**

Ten carers of a loved one diagnosed with autism and anorexia nervosa were interviewed using a semi-structured interview schedule and the transcripts were analysed with thematic analysis.

**Results:**

Four key themes emerged from the interviews: the role of autism in anorexia nervosa, carers’ problems with clinical services, the impact on carers and suggestions for future improvements.

**Conclusions:**

Carers agreed that autism played a significant role in the development and maintenance of their daughters’ anorexia nervosa. However, this comorbidity does not appear to be appropriately addressed in current treatment provisions. They described several difficulties, including problems getting an autism diagnosis and the perception that eating disorder services did not accept or adapt around the condition. This resulted in feelings of frustration and isolation for families, a scenario exacerbated by a perceived lack of support or specific resources for carers of individuals on the autism spectrum. Clinical recommendations on the basis of the current and previous studies are outlined.

## Background

The question of whether autism and anorexia nervosa might be linked was highlighted in the research literature as early as 1983,^1^ with case studies presented in this area as early as 1988.^2^ Autism is a neurodevelopmental condition that is associated with difficulties in social functioning, communication and restricted interests and patterns of behaviour.^3^ Anorexia nervosa is a psychiatric disorder characterised by low body weight, fear of gaining weight and severe weight and shape concerns.^3^ According to epidemiological studies anorexia nervosa is a disorder that largely affects women, with estimates ranging from 3:1 to 18:1 female to male ratios, and autism is a condition that largely affects men with a 4:1 male to female ratio.^4–7^ Women are also more likely to be identified and diagnosed much later in life than men, potentially because of a different clinical presentation that is missed by standard assessment tools.^8,9^ There is a growing awareness that previous autism research is male biased, and there have been calls for more research to improve the understanding and recognition of autism in women.^10^

## Challenges raised by comorbidity

Although autism and anorexia nervosa represent separate conditions, evidence suggests a number of similarities between these two diagnoses, particularly in the area of cognitive rigidity.^11^ In the light of these similarities, various studies conducted in eating disorder settings have examined the prevalence of autism among populations with anorexia nervosa using different tools to measure autistic traits, with estimates ranging from 8 to 37%.^12–14^ These findings are replicated in adolescent patients suggesting that adolescents with anorexia nervosa also have elevated autistic features.^15^ In patients with anorexia nervosa, higher levels of autistic traits are associated with poorer treatment outcomes, more severe presentations and a longer length of stay in in-patient settings.^16,17^ This group of patients also appear to respond to specific elements of treatment differently, for example showing little clinical change after group psychology interventions^18,19^ but showing significant improvements after the same intervention delivered in individual formats.^19^ Alternatively, autistic traits may be associated with protective factors in treatment: people with autism who have anorexia nervosa may exhibit higher levels of treatment adherence compared with those with anorexia nervosa only.^12^ These findings suggest that people with autism may be responding to elements of treatment differently and could benefit from adaptations to the standard anorexia nervosa treatment pathway.

However, there are currently no guidelines for adapting anorexia nervosa treatment for people with autism. In the UK, the National Institute for Health and Care Excellence (NICE) guidelines for Eating Disorders [NG69] has no mention of co-occurring neurodevelopmental disorders, although it does have guidance on physical and mental health comorbidities.^20^ Recent qualitative studies interviewing clinicians and patients suggest a clear need for treatment adaptations.^21,22^ These studies suggest that although the role of autism in anorexia nervosa needs to be considered during treatment, and appropriate adaptations made accordingly, at present clinicians commonly lack confidence or training in applying these adaptations. However, no research has yet explored the views of carers in this area, despite carers often being expected to be involved in eating disorder treatment.

## Aims

Studies suggest that caring for someone with an eating disorder can be difficult for carers, and is often associated with a perceived lack of resources, support and recognition.^23,24^ To date, no studies have examined how this experience may be affected if the carer is supporting someone with both anorexia nervosa and co-occurring autism. The aim of this study was to explore carers’ experiences of current treatment approaches for people with autism who have anorexia nervosa, and their views on how these can be improved. Alongside recent stakeholder interviews with patients and clinicians^21,22^ we were particularly interested if there was any overlap with the emerging themes from these previous studies. Integration of these interviews will aid understanding of current treatments for people with autism who have anorexia nervosa and inform future studies on how to adapt treatment to better serve these individuals and their carers.

## Method

Semi-structured interviews were conducted with carers who have current or historic caring responsibilities for an individual with both diagnosed autism and anorexia nervosa. Written informed consent was obtained from all participants. The authors assert that all procedures contributing to this work comply with the ethical standards of the Helsinki declaration of 1975, as revised in 2008. Ethical approval was obtained from London-City and East Research Ethics Committee and South London (18/LO/0050).

### Recruitment

Participants were recruited through social media, from contact with the study site through previous research and were approached by the principal investigator's (K.T.) contacts. Participants were considered eligible if they had or have had caring responsibility for someone with diagnoses of both autism and anorexia nervosa. The recruitment process continued until all authors agreed data saturation had been reached. After six interviews all authors read the full transcripts and met to agree initial themes, and subsequently met until all authors agreed that saturation had been achieved. Data saturation was judged as the point at which no new information was felt to be emerging from the interviews.

### Data collection

Carers were interviewed via skype, phone or in person by author J.A. with interviews lasting between 40 and 60 min. Four carers were interviewed on skype, five via phone call and one in person. Interviews followed a semi-structured format with pre-agreed questions, agreed by the study team and based on questions from previous clinician and patient interviews.^21,22^ An example of the types of questions asked were: ‘Did you/your loved one experience difficulties accessing clinical services because of co-occurring autism?’ and ‘Do you feel like the autism was taken into account in previous treatments?’. The full interview schedule is available in supplementary File 1 (available at https://doi.org/10.1192/bjo.2020.36).

### Participant characteristics

In total, ten carers completed the interviews. Nine of the participants were mothers to a daughter with both conditions, and one participant was a father. Participants’ daughters’ ages ranged from 16 to 25 with an average length of an autism diagnosis of 4.9 years and anorexia nervosa diagnosis of 4 years. Three were diagnosed with anorexia nervosa prior to their diagnosis of autism and all had at least one other comorbid diagnosis with the most common being obsessive–compulsive disorder (OCD) and general anxiety disorder. The average age of autism diagnosis for the daughters in this sample was 15 years (9–23 range), this is in the context of a UK national median diagnosis age of 55 months.^25^ Nine carers were from different parts of the UK and one carer was from the USA.

### Analysis

All interviews were recorded live with Dictaphone software and transcribed. J.A., K.T., M.O. and E.K. individually read each transcript. Authors then met to agree a deductive coding framework based on the research aims and findings from previous research in this area. Authors then individually coded the data line by line. All authors met on two occasions to discuss the coded data and achieve consensus on the themes. Data was analysed using thematic analysis methodology.^26^ Four themes emerged from the interviews:

- the role of autism in anorexia nervosa;
- problems with services;
- impact on carers;
- and improvements.

## Results

**Fig. 1:**
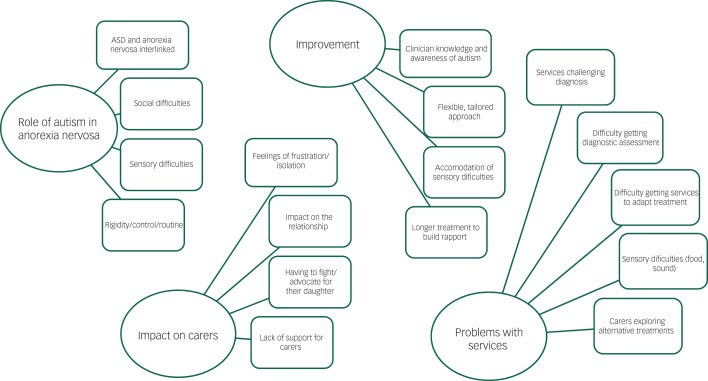
Main themes and subthemes results from thematic analysis.

The main themes and subthemes are summarised in the thematic map, Fig. 1. Themes and subthemes are summarised under their theme headings.

ASD, autism spectrum disorder.

### Role of autism in anorexia nervosa

All carers felt that the anorexia nervosa experienced by their daughters was closely interlinked with their autism, rather than representing separate conditions. They described multiple trait crossovers such as cognitive rigidity, attention to detail and routine behaviours. Carers identified overlaps in behaviours associated with autism and anorexia nervosa, but felt that the mechanisms underlying the behaviours were potentially different for their daughters with autism compared with people with anorexia nervosa only. For example, cognitive rigidity and routine behaviours were identified as existing prior to anorexia nervosa onset, but then worsening with illness development and weight loss.

Autism was also felt to contribute to the development of anorexia nervosa in ways not accounted for in traditional eating disorder formulations. Four carers (40%) believed that anorexia nervosa potentially acted as a coping mechanism for difficulties associated with autism. Similarly, eight carers (80%) identified social difficulties associated with autism as contributing to the development of anorexia nervosa. Many of the carers mentioned that their daughters had the ability to mask their difficulties and appear to be able to cope in some social settings, but this often became difficult as they got older and social situations became more complex, such as starting university. The emergence of anorexia nervosa appeared to be related to this reduced ability to cope:

‘I realised just how much as a mother and as a family we had been kind of managing her friendships and play dates and I think, as is quite classic as I understand now with female autism especially, that is when really the friends sort of start to drift away, because image is important and the slightly odd friend is not necessarily welcome.’ (Carer 7)

All but one of the carers (90%) described sensory issues complicating the anorexia nervosa symptoms and making it hard for clinicians to ascertain what behaviours were because of autism and what were because of anorexia nervosa. This was particularly apparent around food choices, with many carers highlighting that the standard refeeding programme was difficult as the meals often involved eating foods that they would not normally eat, or they would have difficulties with the sensory aspect of the food. These difficulties were described as existing from a very early age and therefore pre-dating any anorexia nervosa symptoms. The refeeding programmes would be described as problematic when the food options were too rigid and did not take into account any difficulties with certain foods that existed prior to anorexia nervosa onset.

### Problems with services

Carers identified problems with both eating disorder and autism services. Seven carers (70%) described how they had to overcome multiple barriers to get their daughter an autism assessment. Four carers had to pay for a private diagnostic assessment after being turned away from services provided by the National Health Service. Many carers commented on the lack of understanding of female autism in the community and mental health services they approached for assessment, suggesting that either they were not offered an assessment, or received a negative result only to later receive an autism diagnosis.

‘It was absolutely appalling… particularly poor for girls because, as you probably know, they don't present the same as boys. For years they have been diagnosing girls on the boys’ criteria which is hopeless because that is for the boys, not the girls.’ (Carer 1, describing their experience of the diagnostic process)

Furthermore, those that did receive an autism diagnosis felt that this then led to problems accessing eating disorder services, including the perception that eating disorder services challenged or refused to acknowledge the autism diagnosis. This often-created tensions between the family and the care team, creating further issues with therapeutic engagement and carer support.

‘You have to leave the autism at the door, all our girls are like this.’ (Carer 2, quoting clinical service response to her informing them of the autism diagnosis)

Even where services did acknowledge the autism diagnosis, carers felt that the clinical teams were unable to make adaptations to the routine anorexia nervosa treatment in order to accommodate any difficulties.

‘I know she used to zone out in the sessions, and she didn't process what was being said. I asked lots of times for written information. You know, you've got to write it down to explain to her, she has an education, health and care plan; you really need to write it down, she is not processing it and that didn't happen at all, not once.’ (Carer 4, talking about her experience of family therapy)

Often treatment environments, especially in-patient settings, were described as not being autism friendly, making it difficult for the individual and their family to engage in traditional treatment settings. For example, the in-patient settings were described as a sensory overload:

‘So you're overwhelming her senses with food, sensations in her stomach, noise, other people crying, high distress, so everything that's going to make an autistic person have a meltdown and behave in what they say is violent or unacceptable behaviour; that thrashing out meltdown, banging her head to calm herself, that only happens when the senses are overwhelmed and you're more likely to get that situation when you start treating an eating disorder.’ (Carer 2)

Overall, carers frequently described themselves as feeling let down by current treatment options, to the extent that they felt forced to explore alternative treatments or provide treatments themselves at home. Many carers described attending courses, reading books and research papers in order to offer some level of treatment at home. Other carers described finding alternative sources of support, including personal trainers or private dieticians, to create a support system for their daughter that is not being offered in current treatment provisions.

‘It was just better to just throw the book out of the window and go with my instinct.’ (Carer 9)

### Impact on carers

Carers described the impact that caring for their daughters had on their lives. A number of carers described having to leave their job, reducing their hours or working from home in order to better support their daughter. This was felt to have a negative impact on both their own well-being and mental health, and their relationships with their daughters.

‘This is the hardest thing as a mum that I have ever had to deal with… I just don't know what to do. I feel like a complete failure as a mum, it is exhausting.’ (Carer 10)

This is compounded with an apparent lack of specific support for carers from professional services. Although carers have positive comments about carer workshops and support, when they are offered, four carers (40%) mentioned the lack of specific support for autism comorbidity or comorbidities in general. Many carers also criticised the lack of post-diagnostic support for their daughters, describing how they were not able to access autism support services as their daughter did not have an intellectual disability (also known as learning disability in UK health services).

‘They are all set up for what I would call, and I don't know what your typical patient with anorexia is like, but it is not our daughter; she has got complex needs and none of them, you know none of the workshops we attended addressed those extra needs.’ (Carer 10)

All of this led to carers feeling frustrated and isolated from existing support systems. Instead, carers described setting up their own support groups and networks online, often on social media, as a way of supporting each other's specific needs. Furthermore, carers described having to self-advocate in order for their daughters to receive appropriate support and then sharing advice on how to go about this on social network groups.

‘My heart breaks for the people and the non-help they are getting and when people start out on their journey and they are saying “I am not getting much help”. I think wow, here we are eight to ten years later and we are still not getting any help at all. Everything that we have had to do I have had to fight for, and I do, and I will because you do, it is your child.’ (Carer 1, describing her experience of online social media groups)

### Improvement

All carers suggested various ways that services could be more friendly for patients with autism. For example, accommodating sensory difficulties by reducing the noise levels down in services by having soft-close doors and being mindful about clinicians shouting across corridors. Furthermore, carers felt that services could be flexible with treatment provisions to accommodate autistic traits, for example, adapting the diet plan to account for sensory difficulties with various food options.

‘There are a few things, particularly noises are a big issue and I would also say that lighting is a big issue as well. When she goes to her meetings with the eating disorder nurse, when they switch the light on it causes her real problems.’ (Carer 6)

The majority of carers (90%) advocated an increase in clinician awareness and knowledge of autism. Several carers described how their daughter had encountered specific clinicians during treatment who had knowledge of autism, and who were able to make adaptations. This was felt to be instrumental in building a rapport with their daughter and providing good care. However, these stories were limited to specific individuals, rather than representing systematic awareness and adaptations across wider treatment teams and hospital settings.

‘It probably is just about education, is it not? … They are treating it purely as an eating disorder, and I think, I do not know, there needs to be some additional support, I do not think there is a full understanding.’ (Carer 6)

Another important consideration that was mentioned by six carers (60%) was the importance of building a rapport with the individual before expecting them to do any difficult psychological work. A few suggestions were to have the first session doing a joint activity or playing a game, in order to build trust. One positive attribute that was mentioned in a number of the interviews was that the clinicians who took the time to build a rapport made a significant impact in the treatment their daughters received.

‘Building a relationship with her, maybe not in the clinic, before expecting her to go to the clinic and be weighed. So, whether that was by email or by a letter or by asking her if she had any questions, she wanted to ask them but to do it on paper. All of that would have made it easier.’ (Carer 4)

## Discussion

At present, there is limited knowledge about how to provide the best clinical care for people with autism who have anorexia nervosa. The goal of this study was to explore carer views on how this can be achieved, while triangulating these findings with previous research on the views of clinicians and patients.^21,22^ All carers interviewed felt that autism played a significant role in the development and maintenance of their daughters’ anorexia nervosa. However, they commonly perceived that this comorbidity was not appropriately addressed in current treatment provision. This was described as part of a wider problem with available services: participants described several difficulties with service provision, including problems getting an autism diagnosis, and the perception that eating disorder services did not accept or adapt around the condition. This resulted in feelings of frustration and isolation for families, a scenario exacerbated by a perceived lack of support or specific resources for the carers of people with autism. Although interventions and support for people caring for someone with an eating disorder do exist, to date there is no specific support around caring for people with autism who have eating disorders.^27^ In the context of these issues, participants made several recommendations for future improvements and changes.

There are many overlapping themes with interviews previously conducted with people with autism who have anorexia nervosa, and clinicians working in this field.^21,22^ First, all qualitative studies with the main stakeholders identified that there are similarities in the features of both anorexia nervosa and autism, but that the underlying reason for these features might be different. For example, food restriction may be related to sensory sensitivities as well as concerns around weight gain. Furthermore, clinician, carer and patient interviews all highlight the sensory difficulties that people with autism face in current treatment settings that are largely overstimulating and require adaptation.^28^

Another overlapping theme was the need for longer treatment and interventions in order to build a rapport with the individual, and to improve treatment adherence, engagement and outcomes. This reflects previous recommendations from research on improving cognitive–behavioural therapy outcomes for people with autism who have depression, anxiety or OCD.^29^ Significantly, this contrasts with the current climate of shorter in-patient admissions for patients with eating disorders as recommended by NICE guidelines.^20^ Clinical services will need to weigh up the potential benefits and improvements in outcomes with longer admissions for those with both conditions, and the potentially increased risk of institutionalisation that might occur from detaching the individual from their usual support and social networks.

The importance of a flexible and individualised approach to treatment was highlighted in the present study and previous clinicians and patient interviews. A common theme across these studies relates to a lack of awareness and understanding of autism in women, and the role that autism can play in anorexia nervosa.^30^ A lack of understanding might be hindering services ability to adapt and modify treatment programmes effectively to cater for the individual needs of these patients. The study findings fits in with previous literature that has found that the needs of women with autism are not being met in current mental health services.^28,31,32^ Future research should investigate how best to improve clinician awareness and knowledge in this area, in order to facilitate a more flexible approach to current treatment provision. A key area of service provision not meeting the needs of women with autism appears to be that of accessing an autism diagnosis. While this was highlighted by carers in the current study, previous research with clinicians working in eating disorder treatment settings suggest that there is no clear pathway for eating disorder clinicians to refer their patients for an autism assessment.^21^ An additional barrier may relate to whether current assessments are appropriate for women. Recent research suggests that the Autism Diagnostic Observation Schedule may work well in identifying potential high autistic traits in women with anorexia nervosa.^9^ Future studies on diagnostic tools for autism in women could benefit clinicians working in the eating disorder field.

### Strengths and limitations

One limitation with the present study is that 9/10 of the participants were mothers. This may reflect the fact that women are more likely than men to provide care for family members with mental health difficulties.^33^ Additionally, all carers in the study were providing care for their daughter with anorexia nervosa. In treatment settings, women with anorexia nervosa outnumber men with a ratio of around 10:1.^34^ Future research should explore the opinions of fathers and other caring roles, such as partners, to ensure that we have a holistic view of carers needs.

The study also has a number of strengths. This is the first qualitative study exploring carers views on autism and anorexia nervosa comorbidity. The sample for the study came from a large geographical area across the UK and one participant from the USA, which supports real practice evidence for assessing current treatment provisions in the UK. Further research would need to be undertaken before extrapolating these findings outside of the UK, particularly in healthcare settings with different models of care. Despite coming from all over the UK, the carer stories were largely similar, further supporting the validity of the findings. This study also raised new questions about the psychological well-being of families and the potential economic costs for society with many carers disclosing that they have stopped working or were working reduced hours in order to support their loved ones. From this preliminary research, it is clear that carers reported issues that could be more effectively addressed and have clear clinical and research implications. For example, in order to address the lack of specific support around this comorbidity, specific psychoeducation and resources could be developed to address the needs of both patients and families.

### Clinical implications

The current study, combined with previous research in the field, indicates that people with autism who have anorexia nervosa may benefit from treatment adaptations. These suggestions require further evaluation and empirical research to establish their efficacy. Potential avenues for future research on adaptations in this area include the following.

- The provision of more intensive care.^35^
- Individual interventions rather than group work.^18,19,36^
- Allowing for longer treatment durations.^37^
- Training and supervision in working with people with autism for eating disorder clinicians, for example in adapting communicative styles (Kinnaird et al,^21,22^ current study).
- Considering the role of autism in the eating disorder formulation (Kinnaird et al,^22^ current study).
- Accommodating sensory difficulties in nutritional rehabilitation and in the wider treatment environment (Kinnaird et al,^21,22^ current study).
- Provision of support for carers (current study).
- Establish clear pathways for individuals presenting with autistic traits, to be assessed for an autism diagnosis in a timely manner (Kinnaird et al,^21^ current study).

Overall, a key theme identified across the current study and previous qualitative research in this area has been the importance of clinicians taking a flexible and individualised approach when working with this population.^21,22^

## Data Availability

Anonymised data is available upon request.

## Supplementary material

For supplementary material accompanying this paper visit http://dx.doi.org/10.1192/bjo.2020.36. click here to view supplementary material
