# Supplementary material for: Carers’ views on autism and eating disorders comorbidity: qualitative study
Source: BJPsych Open. 2020 May 18;6(3):e51. doi: 10.1192/bjo.2020.36 (PMC7331083; doi:10.1192/bjo.2020.36)
Supplement: Supplementary file 1 [file bjosup.zip › S2056472420000368sup001.docx]

**AN/ASD Interview Schedule - Carers**

| **Question** | **Follow-up questions/ prompts (optional)** |
| --- | --- |
| Does your loved one have a formal diagnosis? | How long have you they been diagnosed with AN?  When were they diagnosed with ASD? |
| Is this their first time in treatment? | What treatment have you they had before?  Specify: out/in patient, therapy, medication etc. |
| Did you/loved one experience difficulty accessing services because of co-occurring ASD? |  |
| Do you feel like the ASD was taken into account in previous treatments? | Where treatments modified?  If so – what were the modifications? |
| What is your experience (carers) of the diagnostic process for ASD |  |
| Do you think someone with AN/ASD has additional support needs? | Were they being met?  What could be done differently to meet these needs? |
| What do you feel has been good about their current/previous treatment? |  |
| Have there been any problems or issues you’ve had with treatments offered? | Were these problems resolved?  Did you tell anyone about these problems?  How did this affect your engagement with treatment? As a carer  Why do you think these problems occurred? |
| How do you think treatment for people with AN/ASD could be improved? | Is there anything not currently offered that you think could be particularly helpful for you as a carer?  Is there anything not currently offered that you think could be particularly helpful to them as a patient?  Is there anything currently offered that you’d like to have more of? |
| Do you think there’s a difference between the kind of treatments required by people with AN/ASD, and people with AN only? | Why not?  Why do you think these differences exist? |
| Do you feel like clinicians have adapted the treatment for your ASD? | How have they done this?  How well did they do this? |
| How would you define recovery? | How do you view the relationship between recovering from AN and/or ASD? |
| Do you feel involved in the treatment process? | What works?  What can be changed?  Did/do you feel listened to? |
| Any further thoughts? |  |
